# Magnetic resonance imaging T2^*^ of the pancreas value using an online software tool and correlate with T2^*^ value of myocardium and liver among patients with transfusion-dependent thalassemia major

**DOI:** 10.3389/fradi.2022.943102

**Published:** 2022-09-06

**Authors:** Han Guan Hoe, Kim-Ann Git, C-Khai Loh, Zarina Abdul Latiff, Joyce Hong, Hamzaini Abdul Hamid, Wan Noor Afzan Wan Sulaiman, Faizah Mohd Zaki

**Affiliations:** ^1^Department of Radiology, Universiti Kebangsaan Malaysia Medical Center, Kuala Lumpur, Malaysia; ^2^Department of Radiology, Hospital Selayang, Batu Caves, Malaysia; ^3^Paediatric Oncology and Haematology Unit, Department of Paediatrics, Universiti Kebangsaan Malaysia Medical Center, Kuala Lumpur, Malaysia; ^4^Paediatric Endocrinology Unit, Department of Paediatrics, Universiti Kebangsaan Malaysia Medical Center, Kuala Lumpur, Malaysia

**Keywords:** thalassemia, MRI T2^*^, haemosiderosis, diabetes mellitus, pancreas

## Abstract

**Objective:**

Patients with thalassemia major do require lifetime blood transfusions that eventually result in iron accumulation in different organs. We described the usefulness of using magnetic resonance imaging (MRI) T2^*^imaging values for the evaluation of pancreatic iron load in these patients, and we correlated it with MRI T2^*^ haemosiderosis of the myocardium and liver that has been recognized as a non-invasive assessment of iron overload among patients with thalassemia major.

**Materials and methods:**

We conducted a cross-sectional study on 39 patients with thalassemia major in one of the tertiary university hospitals for a 1-year period. Demographic data were collected from the patient's history. MRI T2^*^ of the pancreas, liver, and heart were executed on all patients in the same setting. Objective values of iron overload in these organs were obtained using the MRI post-processing software from online software.

**Results:**

A total of 32 (82.1%) patients had pancreatic iron overload including 2 patients (5.1%) with severe iron overload and 15 patients (38.5%) with moderate and mild iron overload, respectively. Nine patients (23.1%) had myocardial iron overload, which included 3 patients (7.7%) who had severe cardiac haemosiderosis. Notably, 37 patients (94.9%) had liver iron overload, which included 15 patients (38.5%) who had severe liver haemosiderosis. There was a moderate positive correlation between the relaxation time of the pancreas and heart haemosiderosis (r = 0.504, *P* < 0.001). No significant correlation was found between the relaxation time of the pancreas with the liver and the heart with the liver.

**Conclusion:**

Pancreatic haemosiderosis precedes cardiac haemosiderosis, which establishes a basis for initiating earlier iron chelation therapy to patients with thalassemia major.

## Introduction

Thalassemia major is a type of haemolytic anemia. There are many different types of thalassemia, and the most common type is beta thalassemia major, which is characterized by biosynthesis defect of beta-globin chains (1, 2). The mainstay treatment of thalassemia is regular transfusion of the packed red blood cell. Most patients with thalassemia require lifetime transfusions and repetitive transfusions, which result in the accumulation of iron in different organs, such as the liver, heart, pancreas, lungs, endocrine glands, and kidneys, which subsequently causes the failure of these organs (1, 2).

Advances in B-thalassemia major management have improved survival. Relatedly, it is paramount to have methods of non-invasive iron overload estimation in different organs of patients with thalassemia. In the past, serum ferritin had been utilized as a marker for iron store evaluation, but conditions such as inflammation, infection, liver disease, or hepatitis can raise ferritin levels and, thus, it is a non-specific marker for iron storage and illness in general (1–4). Although biopsy is considered the gold standard for estimating iron overload in the organ, it is invasive and has potential complications such as bleeding, so the practice is limited (4).

The T2^*^ magnetic resonance imaging (MRI) technique is a valid, accurate, and non-invasive method for the evaluation of tissue iron storage. This method has revolutionized thalassemia patient management, mainly in modifying iron chelation regimens (5–7). Iron concentration in the liver is a suitable index for total body iron, but various mechanisms of iron loading result in different mechanisms of iron uptake and excretion between the liver and the other organs. It is proven that cardiac dysfunction is the sequel of cardiac iron overload (6). MRI screening of liver and heart iron has become part of standard care in major thalassemia centers (8). MRI vendors have provided in-house T2^*^ value measurement that has been validated from previous research (8, 9) and has been practiced regularly in our institution.

However, there has been controversy regarding the post-processing technique of T2^*^ with its accuracy allegedly affected by signal-to-noise ratio, noise distribution, and artifacts, among others (10). Hence, few other options were proposed in the literature to utilize an automated truncation method to reduce post-processing pitfalls, especially in measuring severe iron overload (10–12). Online software using such a method has been developed and it is compatible with most web browsers with the web address of http://www.isodense.com/ic. It is an open-source platform for T2^*^ value calculation, and it is comparable with the standard reference software (12).

The pancreas is one of the known solid organs that can be affected by iron overload. Thus, the pancreatic function in regulating glucose metabolism by insulin production can be affected, which has been observed among patients with thalassemia (13). There have been scanty papers demonstrating the complication of hyperinsulinemia among patients with thalassemia who were diagnosed using MRI T2^*^ pancreas (14).

This study is aimed to determine the value of using MRI T2^*^ imaging in evaluating pancreatic iron load among patients with transfusion-dependent thalassemia, and we seek to correlate T2^*^ values between relaxation time of the liver, myocardium, and pancreas among patients with thalassemia major in MRI T2^*^ using the aforementioned online software tool.

## Materials and methods

### Study design and subject recruitment

This was a cross-sectional and prospective study, conducted in one of the tertiary university hospitals for a 1-year period. The study was approved by the hospital ethics committee with ethic approval number FF-2017-122. The sample size needed was at least 36 samples to obtain 80% power and for the detection of correlation as small as 0.45. This study included 39 samples whereby informed consent was obtained. Inclusion criteria included (1) all patients were those with thalassemia major who received regular blood transfusions at 2-to-4-week intervals for at least 18 months prior to research participation, (2) received iron chelation therapy, (3) had regular serum ferritin with the most recent is within 1 week before or after the MRI pancreas, and (4) verbal and written consent obtained prior to the study from the patients and the parents for the pediatric patients.

The exclusion criteria especially for the pediatric population included (1) patients who were younger than 5 years old (since they would not receive enough transfusion), (2) patients who required general anesthesia or sedation for the MRI scan, (3) patients who were uncooperative, (4) patients who had absolute or relative contraindications for MRI (e.g., cardiac valve replacement, insulin pump, and cochlear implant), and (5) those who were claustrophobic. Criteria 4 and 5 are also applicable to the adult population.

### Methods

Patients who fulfilled the inclusion criteria were recruited from the hospital paediatric day care center and then arranged for an MRI T2^*^ scan appointment. The MRI was performed in a 1.5 Tesla Siemens Avanto MRI scanner (Erlangen, Germany) using a standard body coil. The MRI scans were synchronized to the cardiac cycle using a standard ECG gating. The T2^*^ gradient echo pulse sequence was obtained in 3 specific regions [i.e., heart (short axis of the left ventricle), liver, and pancreas]. MRI cardiac T2^*^, liver, and pancreas were performed with the breath-hold technique. The T2^*^ technique for the heart, liver, and pancreas was as follows: heart (bright blood) – slice thickness: 10 mm; field of view (FOV): 400 mm; flip angle: 20; matrix: 256 × 128; TE: 2.0 to 3.0 minimum; increment from 1.5 to 2.0 ms; up to 16 ms; number of TEs: 10; bandwidth: 810 Hz/pixel; flow compensation: on; fat saturation: off; heart (dark blood)–other parameters were similar except for the flow compensation (off) with extra double inversion pulse and TI to extend to diastole; liver–slice thickness: 10 mm; FOV: 400 mm; matrix: 96-128 × 64-96; flip angle: 20; and time to echo (TE): ideally 0.8–1.3 ms (minimum), increments (echo spacing) of 0.8 to 1.0 ms; up to 12 ms; number of TEs: 10; bandwidth: 810 Hz/pixel; flow compensation: on; fat saturation: off; TR: 100–150 ms. In contrast, the T2^*^ pancreas technique adopted similar to the T2^*^ liver technique except for the reduced slice thickness of 6 mm. We imaged the pancreas in axial cuts at the pancreatic neck and body where we could identify the consistent anatomy, i.e., confluence of splenic vein and superior mesenteric vein (portal venous confluence). We discovered that selecting the region of interest (ROI) at the pancreatic neck or proximal pancreatic body would produce the most consistent result because this part of the pancreatic parenchyma is less likely to be atrophied. In addition, there is less motion artifact from the adjacent small bowel loops or adjacent vessels pulsating artifacts and there is more pancreatic parenchyma for the purpose of drawing ROI.

The whole MRI T2^*^ examination (e.g., liver, heart, and pancreas) took ~15 to 20 min per patient wherein having an extra pancreas scan would take an additional 1 to 2 min relative to the usual routine scan.

### Image processing and data analysis

Post-processing of these images was performed with an online open-source tool that has been validated against CVI 42 version 5.1 (Circle Cardiovascular Imaging, Calgary, AB). By using the online open-source automated truncated software, we drew ROIs to allow T2^*^ calculation at the interventricular septum (excluding cardiac vein), liver (excluding obvious portal vein or hepatic veins), and pancreas (excluding splenic artery and vein). This software calculates T2^*^ in an ROI-based algorithm. The mean signal intensity of the ROI was measured for each image and, subsequently, was plotted against the TE. A mono-exponential trendline was incorporated with an equation in the form: y = Ke–TE/T2^*^, where y represents the image signal intensity, K represents a constant, and TE represents the echo time.

The pixel-based approach used an in-house post-processing software, whereby segmented images were imported into the MRI machine commercial software. The signal intensity of each pixel was plotted against the TE. Subsequently, the T2^*^ was calculated by using a mono-exponential equation to the time course of each pixel, and the map image was produced based on the pixel's T2^*^ value. The T2^*^ RGB map of the liver and heart was created for all patients. The T2^*^ map became the guide to choose the ROI homogenously. The mean T2^*^ pixel values in the ROI were obtained for the liver and heart. The results were tabulated using the statistical analysis of data expressed as mean ± standard deviation, median, frequency, and percentage. The normal T2^*^ value of the heart, liver, and pancreas was based on previous literature (14–16).

Correlations between variables were assessed to evaluate the relation of different factors using Spearman's rank coefficient correlation when at least one of the variables showed a skewed distribution. The magnitude of the correlation was defined based on the value of the correlation coefficient. A correlation <0.4, between 0.4 and 0.6, and more than 0.6 were considered weak, moderate, and strong, respectively. We used the Loess method to fit a smooth line through the variable scatter plot.

All statistical analyses were performed using SPSS (IBM SPSS statistics for windows, version 24.0; IBM Corp, Armonk, NY). *P-*values <0.05 were considered statistically significant, while *p-*values <0.001 was considered highly statistically significant.

## Results

### Demographics data

This study included 39 patients that consists of 19 men and 20 women. The mean age was 23.7 years with an age range between 9 and 37 years. The demographic characteristic of patients is shown in Table 1.

**Table 1:** Demographic data and T2* results for the participants.

Age (year)	Mean +- SD	23.7 ± 11.61
	Median (range)	58
	Minimum	9
	Maximum	67
Gender	Male	19
	Female	20
Ferritin (ng/dl)	Mean +- SD	4640.75 ± 4821.93
	Median (range)	19115.08
	Minimum	112.15
	Maximum	19227.23
	Normal	1
	Abnormal	38
T2*Liver (ms)	Mean +- SD	3.84 ± 3.49
	Median (range)	12.30
	Minimum	0.70
	Maximum	13.00
	Normal	2 (5.1%)
	Abnormal	
	Severe	15 (38.5%)
	Moderate	11 (28.2%)
	Mild	11 (28.2%)
T2*Heart (ms)	Mean +- SD	32.04 ± 14.94
	Median (range)	51.30
	Minimum	2.60
	Maximum	53.90
	Normal	30 (76.9%)
	Abnormal	
	Severe	3 (7.7%)
	Moderate	6 (15.4%)
	Mild	-
T2*Pancreas (ms)	Mean +- SD	18.28 ± 13.98
	Median (range)	49.10
	Minimum	1.4
	Maximum	50.50
	Normal	7 (17.9%)
	Abnormal	
	Severe	2 (5.1%)
	Moderate	15 (38.5%)
	Mild	15 (38.5%)

### T2^*^ heart, liver, and pancreas

**Figure 1:**
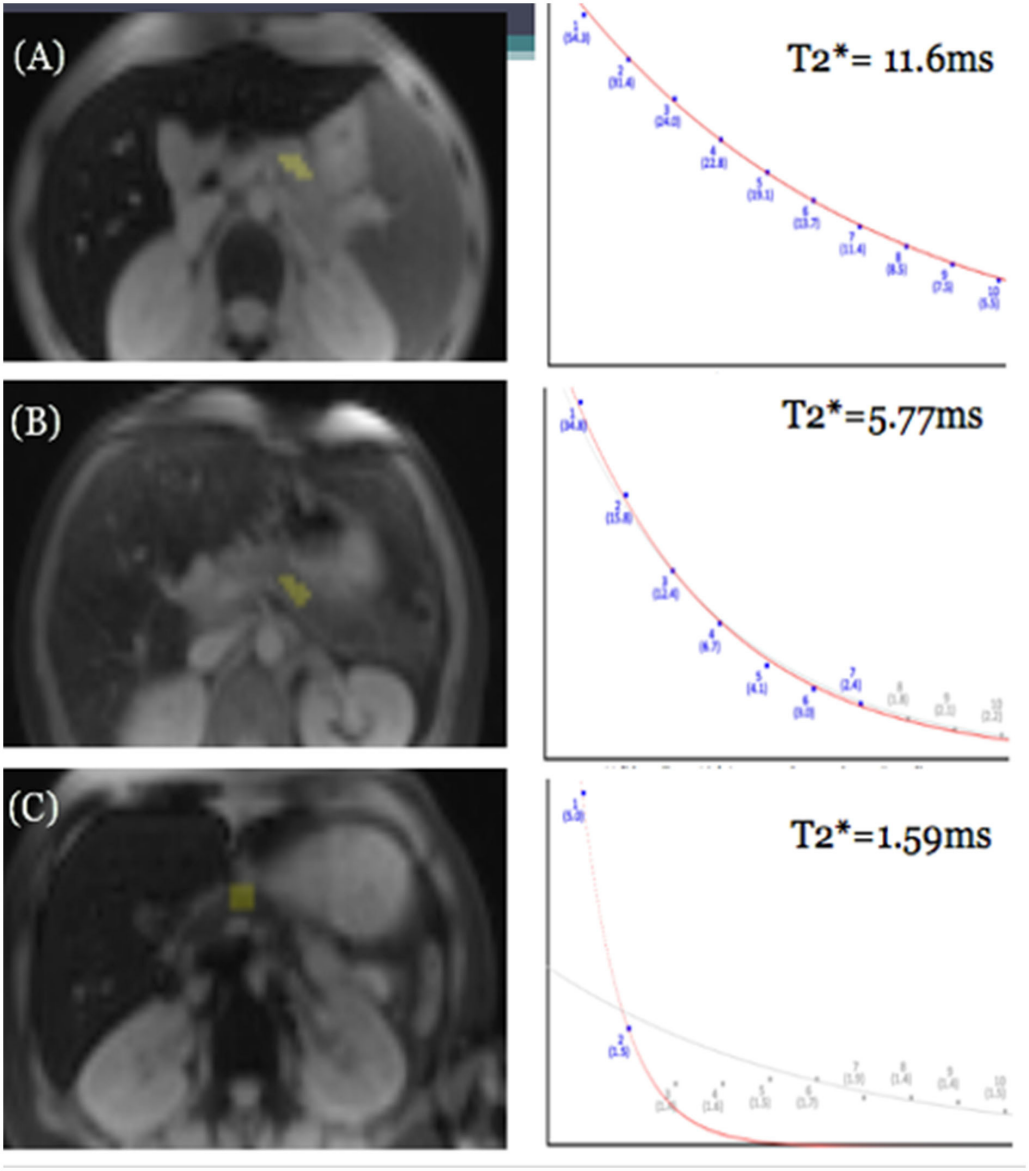
GRE MRI images of three different patients showing mild **(A)**, moderate **(B)**, and severe **(C)** iron deposition in the pancreas at the level of the splenic vein, and corresponding differences in rate of signal decay resulting in different pancreatic T2* values in patients with mild, moderate, and severe pancreatic iron overload.

**Figure 2:**
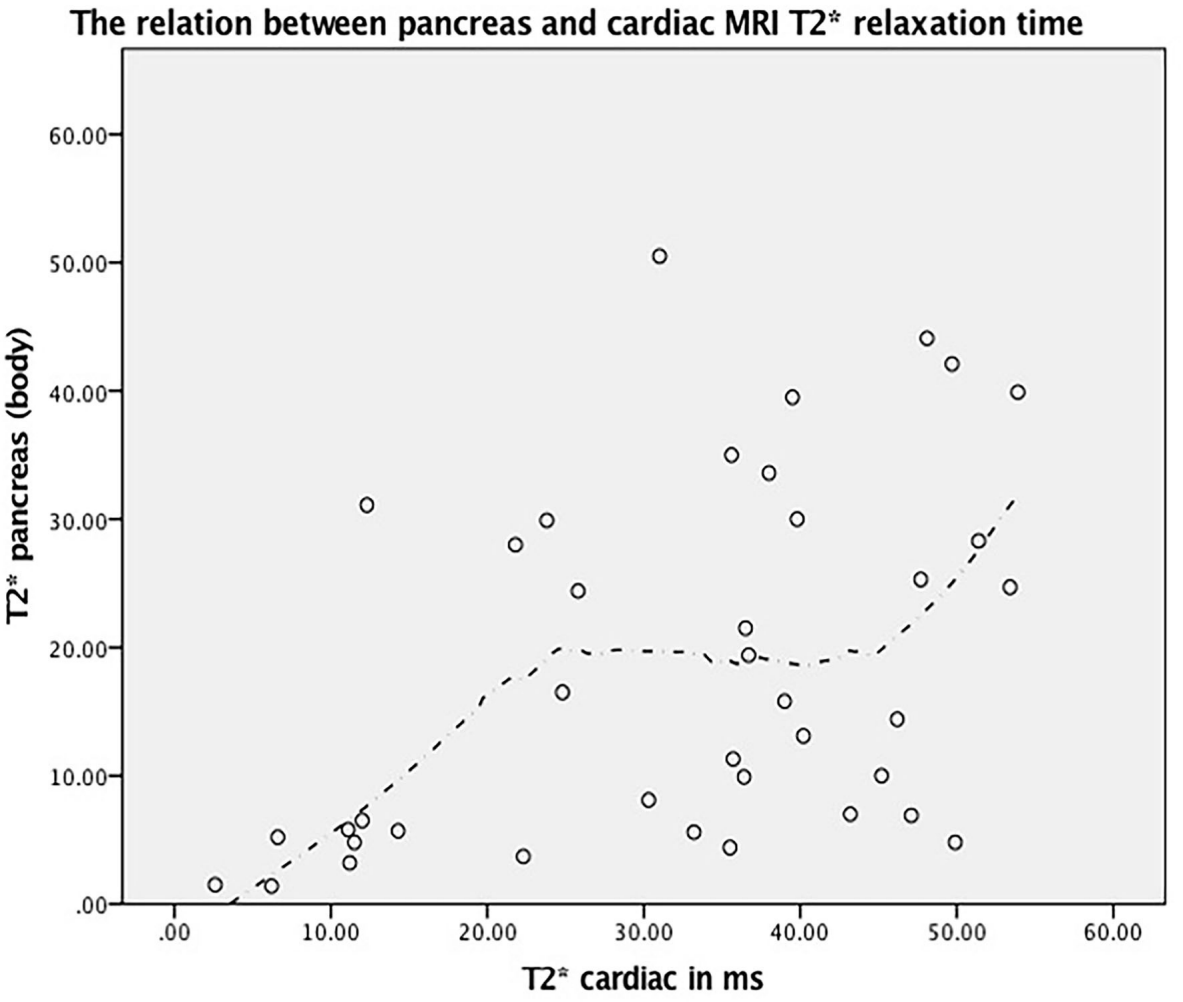
The relation between pancreas and cardiac MRI T2* relaxation time (r = 0.504, *P* < 0.001) and its corresponding fitted line based on Loess method.

The mean relaxation time of the liver, pancreas, and heart were 18.28 ± 13.98 ms, 32.04 ± 14.94 ms, and 3.84 ± 3.49 ms, respectively. A total of 32 (82.1%) patients had pancreatic iron overload, including 2 patients (5.1%) who had severe iron overload, 15 patients (38.5%) who had moderate iron overload, and 15 patients (38.5%) who had mild iron overload (Figure 1). Notably, 9 patients (23.1%) had myocardial iron overload, including 3 patients (7.7%) with severe cardiac haemosiderosis, 6 patients (15.4%) with moderate cardiac haemosiderosis, and none have mild cardiac haemosiderosis. Of note, 37 patients (94.9%) had liver iron overload, including 15 patients (38.5%) who had severe liver haemosiderosis, and 11 patients (28.2%) had both moderate and mild iron overload. The findings related to serum ferritin, heart, liver, and pancreas MRI T2^*^ are summarized in Table 2. There was a strong negative correlation demonstrated between serum ferritin and relaxation time of liver T2^*^ (r = −0.734, *P* < 0.001), and a weak correlation was seen between serum ferritin and cardiac relaxation time (r = −0.344, *P* < 0.05) and a moderate negative correlation between ferritin and pancreas relaxation time (r = −0.401, *P* < 0.05). When comparing between the relaxation time of liver, pancreas, and heart T2^*^, there was a moderate positive correlation between the relaxation time of pancreas and heart (r = 0.504, *P* < 0.01) (Figure 2). However, there was no significant correlation between the relaxation time of the pancreas with liver and the heart with liver (Table 2).

**Table 2:** The spearman correlation between T2^*^values.

**First parameter**	**Second parameter**	**r**	***P-value***
**T2** ^*^ **value parameter 1**	**T2** ^*^ **parameter 2**	**r**	***p-value***
T2^*^ Pancreas	T2^*^ Liver (By online tool)	0.270	0.096
	T2^*^ Heart	0.504	<0.001^*^
T2^*^ Heart	T2^*^ Liver (By online tool)	0.223	0.172
T2^*^ Heart by online tool	T2^*^ heart by pixel based software	0.789	<0.001^*^
T2^*^ Liver by online tool	T2^*^ liver by pixel based software	0.595	<0.001^*^
Mild liver haemosiderosis T2^*^ Liver by online tool	T2^*^ Liver by pixel based software	0.951	<0.001^*^
Moderate liver haemosiderosis T2^*^ Liver by online tool	T2^*^ Liver by pixel based software	0.433	0.184
Severe liver haemosiderosis T2^*^ Liver by online tool	T2^*^ Liver by pixel based software	0.242	0.385
Serum ferritin	T2^*^ Liver (By online tool)	−0.734	<0.001^*^
	T2^*^ Liver (by color RGB	−0.485	<0.001^*^
	map)		
	T2^*^ Heart	−0.344	<0.05
	T2^*^ Pancreas	−0.401	<0.05

* p <0.001; statistically significant.

### T2^*^ value using online open-source software and color RGB map tool

The mean T2^*^ value for the liver was 3.84 ms for the online automated truncated open-source software and 5.45 ms for the color RGB map tool. The mean T2^*^ value for the heart was 27.67 ms for the online tool and 32.04 ms for the color RGB map tool. In the liver, the mean difference was 1.608 ms (95% CI 0.963 to 2.252 ms) with a correlation coefficient of 0.595 (moderate correlation), while in the heart, the mean difference was 4.367 ms (95% CI 1.559 to 7.174 ms) with a correlation coefficient of 0.782 (strong correlation) (Table 2).

Whenever the online tool showed severe liver iron content, the color map software tended to provide a higher T2^*^ value, resulting in a mild or moderate haemosiderosis categorization for the patient. Correlation coefficients for T2^*^ values measured between the automated truncated online software and the color RGB map software were 0.951 (strong for mild iron load, *P* < 0.05), 0.433 (moderate for moderate iron load, *p* > 0.05), and 0.243 (no correlation for severe iron load, *p* > 0.05) (Table 2).

These results indicated that there was a marked difference in the mean T2^*^ values derived from the two software for both liver and heart iron content, which results in a significant difference in iron content categorization, especially for the liver iron, as the T2^*^ value range among different categories was small.

## Discussion

T2^*^ MRI has become the technique of choice for the non-invasive quantification of both hepatic and myocardial iron overload. It shows a good sensitivity when compared with the liver iron content of liver biopsies, while cardiac T2^*^ correlates inversely with cardiac iron concentration (6–9).

However, body tissues possess different iron deposition kinetics. Thus, it would be useful to have information on the iron overload of other organs, especially the pancreas, since many patients with thalassemia have diabetes as a long-term complication of regular blood transfusion (2, 3) as a result of iron deposition within the pancreatic tissue. The T2^*^ MRI technique for the quantification of pancreatic iron overload is less studied in previous research and has not been studied in our population (14, 17–19).

Cumulative iron-mediated toxicity intensified by the natural process of aging makes diabetes in patients with thalassemia major a significant clinical problem. Pancreatic iron loading in patients with thalassemia major begins in early childhood, and previous studies have shown the prevalence of diabetes mellitus in these patients ranging from 10.4 to 19.5% (19).

The majority of our patients with thalassemia [32 out of 39 patients (82.1%)] showed pancreatic haemosiderosis, while only 9 (23.1%) patients showed cardiac haemosiderosis categorized between moderate to severe cardiac haemosiderosis. Interestingly, they had similar categories for their pancreatic haemosiderosis, for example, those who had severe pancreatic haemosiderosis also had severe cardiac haemosiderosis to a moderate degree. The similar degree of iron overload severity in both the heart and pancreas prove that the pancreas, like the heart, exclusively loads non-transferrin-bound iron (NTBI). The kinetics of pancreatic iron loading and unloading are intermediate between the heart and the liver, making the pancreas T2^*^ a better predictor of cardiac iron than liver iron (14). This study is concordant with previous studies that showed a prevalence of 80% and 80.4% of pancreatic haemosiderosis (14, 17).

This study demonstrated that patients with pancreatic haemosiderosis are affected more than those with cardiac haemosiderosis, which suggests that the pancreatic T2^*^ preceded cardiac T2^*^ iron loading. This scenario provides an earlier iron therapeutic window for clinicians to intensify the iron chelating medication to prevent diabetes and, in the longer prospective, prevent cardiac failure. This study concurs with Noetzli et al. (14) who described the possibility of predicting cardiac overload almost one decade in advance by studying pancreatic haemosiderosis in patients with thalassemia. Pancreatic haemosiderosis precedes cardiac haemosiderosis, so by having an earlier window for iron chelating intensification therapy, we hope to make subsequent cardiac failure infrequent for this patient population.

There was moderate agreement between pancreas and cardiac T2^*^ that showed r = 0.504 (*P* < 0.001). This is explained by the fact that both pancreas and cardiac iron overload share the same iron loading pathway. This finding is similar to other study findings (14, 17, 18, 20, 21). One large multicentric observational study indicated that pancreatic iron is a powerful predictor of not only glucose metabolic dysfunction but also the cardiac iron and its complication (22). There was no correlation between MRI T2^*^ pancreas and liver and between cardiac and liver. This finding agrees with previous studies (20, 23), which suggested that using MRI T2^*^ liver relaxation time to predict pancreas iron overload might not be reliable. In other words, liver haemosiderosis is a poor predictor of pancreatic haemosiderosis. In this study, we noticed that drawing the ROI at the pancreatic neck or proximal pancreatic body was consistently reproducible because this part of the pancreas is easily identified by looking for the confluence of splenic and superior mesenteric veins. The other parts of the pancreas (uncinate process, head, distal pancreatic body, and tail) have different parenchymal sizes and anatomical positions between participants. Furthermore, in participants who have pancreatic atrophy, the pancreatic neck and proximal body still have considerable parenchyma for ROI to be drawn and, thus, are suggested to be a standard ROI during the sampling of the T2^*^ measurement.

There was a strong correlation between liver T2^*^ and ferritin that is in line with previous studies (21, 23–25). A weak correlation was identified between cardiac T2^*^ and ferritin, while a moderate correlation occurred between pancreatic T2^*^ and ferritin. Serum ferritin is good for liver iron estimation. However, it does not show a good correlation between myocardial and pancreas T2^*^ (17). Since patients with thalassemia are at a higher risk of having cardiac failure and diabetes mellitus, monitoring serum ferritin only would lead us to miss the critical window period for intensifying the iron chelating medication before developing these severe complications.

However, several studies have also indicated that serum ferritin alone is not the best indicator of iron overload in the organ, especially the heart (26, 27). This finding is supported by the fact that serum ferritin role in redistributing iron during inflammation and the correlation of serum level and deposited total iron in the organs are weak theories (26). At the same time, the early detection of iron overload using MRI T2 star has been emphasized (26, 27).

**Figure 3:**
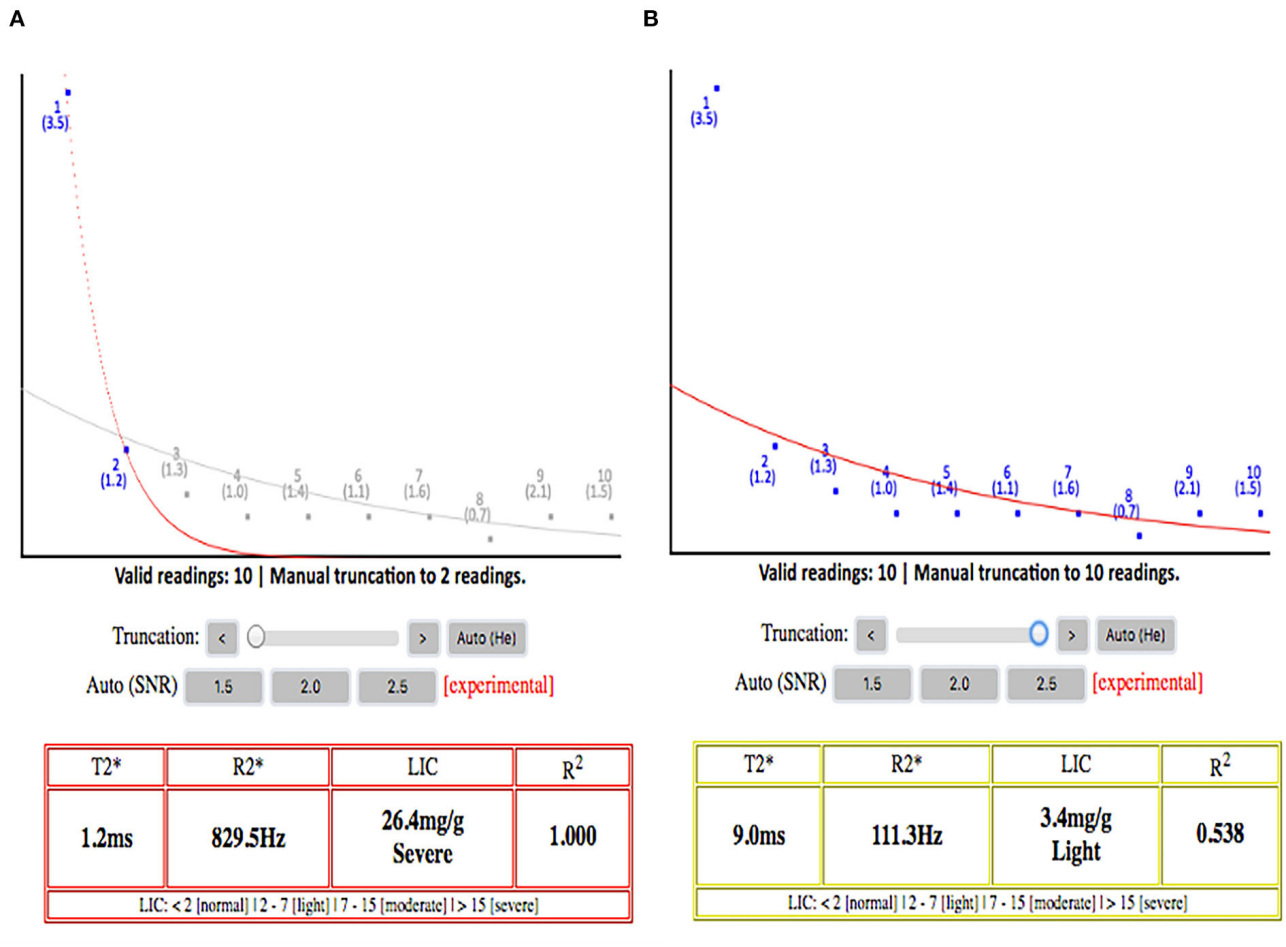
**(A)** Signal decay curve with truncation applied. **(B)** Signal decay without truncation applied. It shows the paramount importance of applying truncation while calculating the T2* because without the truncation, this patient will be wrongly classified as having mild hepatic iron load, while the liver iron load is severe. This will affect the clinician greatly in titrating the iron chelation regimes. The plateau part of the curves on the right side without automated truncation is mainly representing the background signal noises rather than real signal intensities from the liver.

We also found thought-provoking results when comparing the two software (vendor incorporated color RGB map and online software) to quantify T2^*^iron content. There was a significant error in counting the T2^*^ value when using the color RGB map as the ROI to be drawn would include a significant background noise value with increasing TE, especially in those patients with severe iron load. This observation gathers the importance of having automated truncation or taking the extra steps to remove background noise images from the final T2^*^ value calculation (Figure 3). If the radiologist notes a dark liver in signal at the beginning of TE value, they should only use a few initial images on the short TE and omit the longer TE images to avoid inclusion of background noise into the final T2^*^ value calculation; the latter results in overly high T2^*^ values (28, 29).

The automated truncated T2^*^ online tool is a more user-friendly software for estimating iron content, especially for severe iron load, relative to the commercial color RGB map pixel-based approach. Although both software can give approximately similar results, the latter software involves tedious work to manually omit the background noise values at higher TE values (12, 30). This increases the radiologists' workload and the chance of making a mistake in categorizing the patients' iron load. The wrong categorization of iron overload groups is more likely to occur for the liver as the T2^*^ value range for each liver haemosiderosis category is close to each other and small in their value ranges, in comparison with the pancreas and the heart. Therefore, a small increase in the T2^*^ value would result in different categories of liver haemosiderosis when using these two software.

## Study limitations

This study's limitations include the lack of gold standard biopsy data to verify the pancreatic MRI T2^*^ signal. The pancreatic T2^*^ measurements are more difficult than the liver or cardiac T2^*^ because the pancreas is an irregularly structured gland with a variable course. The splenic vein and portal venous confluence can be a useful landmark but this landmark is not present in splenectomised patients. In addition, bowel gas can artificially lower the T2^*^ values in the pancreas but never <10 ms; this is a reason why 10 ms is a better threshold for significant pancreatic iron overload. Older patients also had a significant fatty replacement with high iron concentrations and gland involution over time makes it harder to define the gland boundaries.

## Conclusion

T2^*^ relaxation time for the pancreas in addition to the heart and liver during the routine annual MRI T2^*^ scanning has proven to be useful for regularly transfused patients with thalassemia major without actually increasing the MRI scanning time by more than 5 min. Pancreatic haemosiderosis precedes cardiac haemosiderosis. The online open-source software is useful and user-friendly and is able to provide reliable T2^*^ methods for quantifying organ iron deposition.

## Data availability statement

Data are available on request due to privacy or other restrictions.

## Ethics statement

The studies involving human participants were reviewed and approved by Research Ethics Committee, Universiti Kebangsaan Malaysia. Written informed consent to participate in this study was provided by the participants' legal guardian/next of kin.

## Author contributions

HH: conceived the presented idea and planned and executed the study. K-AG: conceived the presented idea and planned the study. C-KL, ZA, and JH: verified the analytical methods and clinical advice. WW: coordinate and perform the examination. HA: verified the analytical methods. FM: conceived the presented idea, planned and executed the study, supervised the findings of this study, and edited the manuscript. All authors discussed the results and contributed to the final manuscript.

## Funding

This research has been funded by institution research grant FF-2017-122.

## Conflict of interest

The authors declare that the research was conducted in the absence of any commercial or financial relationships that could be construed as a potential conflict of interest.

## Publisher's note

All claims expressed in this article are solely those of the authors and do not necessarily represent those of their affiliated organizations, or those of the publisher, the editors and the reviewers. Any product that may be evaluated in this article, or claim that may be made by its manufacturer, is not guaranteed or endorsed by the publisher.
